# Comprehensive experimental dataset on large-amplitude Rayleigh-Plateau instability in continuous InkJet printing regime

**DOI:** 10.1016/j.dib.2023.109941

**Published:** 2023-12-19

**Authors:** Guillaume Maîtrejean, Mathis Cousin, François Truong, Vincent Verdoot, Frédéric Hugenell, Denis C.D. Roux

**Affiliations:** Univ. Grenoble Alpes, CNRS, Grenoble INP*, LRP 38000 Grenoble, France

**Keywords:** Rayleigh-Plateau instability, Jet of fluids, Drop, Continuous ink jet

## Abstract

The Rayleigh-Plateau instability, a phenomenon of paramount significance in fluid dynamics, finds widespread application in the Continuous InkJet (CIJ) printing process. This study presents a comprehensive dataset comprising experimental investigations of fluid jet breakup phenomena under large-amplitude stimulation conditions using an industrial CIJ print-head from Markem-Imaje. Unlike previous studies, this dataset encompasses a diverse range of experimental conditions, including nine different Newtonian fluids with meticulously measured rheological properties (viscosities, surface tensions and densities). The applied stimulation amplitudes vary from 5V to 45V, representing a substantial span of excitation levels.

The experimental setup captures the intricate dynamics of fluid jets subjected to these varying conditions, producing a rich collection of over 5,000 high-resolution images depicting the breakup phenomena. Each amplitude of stimulation and fluid type yields more than 55 distinct images, providing detailed insights into the evolving jet morphologies.

To ensure the accuracy and relevance of the dataset, all ejection parameters are rigorously documented and included. The dataset thus serves as a valuable resource for researchers seeking to explore the dynamics of large-amplitude Rayleigh-Plateau instability in CIJ printing. Its comprehensiveness and diversity make it particularly suitable for the application of novel machine learning and deep-learning approaches, enabling the study of jet morphological evolution beyond the confines of classical Rayleigh's theory. This dataset holds promise for advancing our understanding of fluid jet dynamics and enhancing the efficiency and quality of CIJ printing processes.

Specifications Table

**Table:** 

Subject	Hydrodynamics
Specific subject area	Multiphase Flow, Jets of fluids, Continuous InkJet
Data format	Raw
Type of data	Image
Data collection	Experimental CIJ setup using a industrial print-head from Markem-Imaje
Data source location	Grenoble Alpes Université, Grenoble, France
Data accessibility	Repository name: Comprehensive Experimental Dataset on Large-Amplitude Rayleigh-Plateau Instability in Continuous InkJet Printing regime Data identification number: 10.17632/9r8w23sy2v.1 Direct URL to data: https://data.mendeley.com/datasets/9r8w23sy2v/1
Related research article	If your manuscript supports a related research article, please cite this article here. If your manuscript is not related to a research article, please delete this entire row. You should **list only one article here**. Please upload a copy of your related research article to your submission.

## 1 Value of the Data


 

• The dataset stands out for its completeness, ensuring a robust collection of data without any gaps or missing information. Rigorous measurements and documentation of all rheological properties of the included fluids enhance the integrity of the dataset, providing a reliable foundation for analysis.
• The dataset systematically focuses on Newtonian fluids in Continuous InkJet (CIJ) printing, offering a comprehensive exploration of disturbance amplitudes ranging from 5V to 45V. This broad coverage enables a thorough examination of the parameter space, contributing valuable insights into the behavior of CIJ jets under diverse conditions.
• An additional distinctive feature of the dataset is its wealth of visual data. For each jet, including different disturbance amplitudes and fluid properties, approximately 55 meticulously captured images are available. These images offer a dynamic and detailed view of the breakup process, serving as a crucial complement to quantitative information.

## 2 Background

The Rayleigh-Plateau instability arises from persistent perturbations in a liquid stream, dynamically perturbed with sinusoidal components that either grow or decay based on the wave number and the original cylindrical stream's radius. Mathematical derivations show that unstable components, growing exponentially over time, result when the product of wave number and initial stream radius is less than unity. Continuous InkJet (CIJ) technology utilizes this instability for precise droplet formation, governed by the nuanced interplay of geometric parameters. While the study of Newtonian fluids in CIJ applications has seen substantial research, it is important to note that previously available datasets lack essential characteristics. These existing datasets frequently suffer from incompleteness in various dimensions, such as missing rheological data, incomplete experimental information, or inadequate coverage of disturbance amplitude ranges. In contrast, the present dataset distinguishes itself by offering a thorough and systematic approach to the study of Newtonian fluids in CIJ printing. Notably, the only publicly available dataset on that topic is numerically generated [1] and, by construction, does not provide the comprehensive, real-world insights offered by the present dataset. The present dataset stands out as an invaluable resource in the field of CIJ research due to its meticulous attention to completeness and systematic coverage of Newtonian fluids and disturbance amplitudes.

## 3 Data Description

**Fig. 1:**
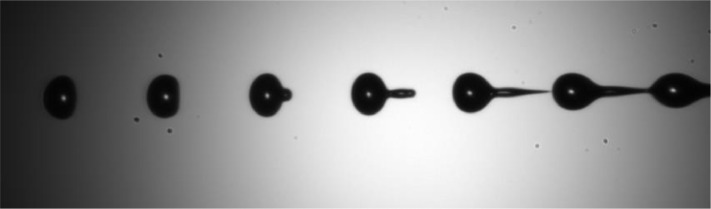
Raw image of a jet breakup named *“*EMKAROX_681_18%_45V _232NOP − 27.png”: 27th image of a jet of a dilution of 18% of EMKAROX 681 in deionized water, stimulated at 45V with a 232 NOP frequency.

The provided dataset comprises a collection of images capturing the dynamic breakup of jetted fluids (see Fig. 1), a detailed description of which is presented in the subsequent section. The dataset organization is designed for easy navigation and reference. Each fluid category is represented by a primary folder with a straightforward naming convention, such as “EMKAROX 130_10%” indicating a 10% mass dilution of EMKAROX in deionized water. Within each fluid category folder, sub-folders are created for various stimulation amplitudes. During the stimulation period, a series of images was captured with minimal dephasing between each successive frame, optimizing the temporal resolution of the breakup process. The duration of the stimulation period is encoded in the file name of each image, quantified in terms of “NOP” (single cycle no operation), where one NOP corresponds to a time interval of 62*.*5*ns*. Additionally, a crucial resource for users is included: a single CSV file named “fluids_properties.csv” residing at the root folder. This CSV file provides comprehensive information about the rheological properties of the fluids, serving as a valuable reference for researchers and practitioners utilizing this dataset for their investigations.

**Fig. 2:**
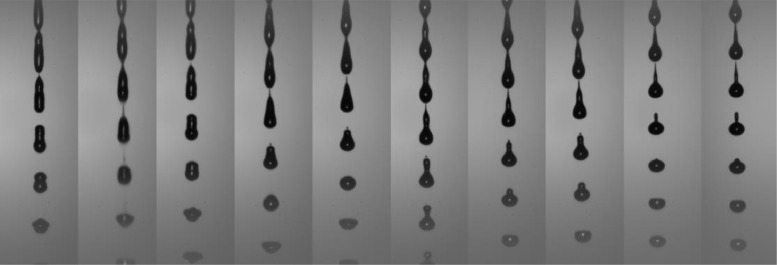
Images of each fluid in the dataset jetted with an amplitude of 45V. From the less viscous (left, deionised water) to the more viscous fluid (EMKAROX 681 18%).

In addition to the images capturing the dynamic breakup of each jetted fluid, an image devoid of any jet presence has been meticulously captured for each fluid category. These “background” images, as indicated by the inclusion of the term “background” in their respective filenames, serve a critical purpose in facilitating the post-processing of the dataset. These background images provide a reference point for subsequent image analysis, aiding in the separation of fluid jet-related features from the overall scene. Fig. 2 provides an illustration of the images with the background subtracted.

## 4 Experimental Design, Materials and Methods

### 4.1 Experimental device

**Fig. 3:**
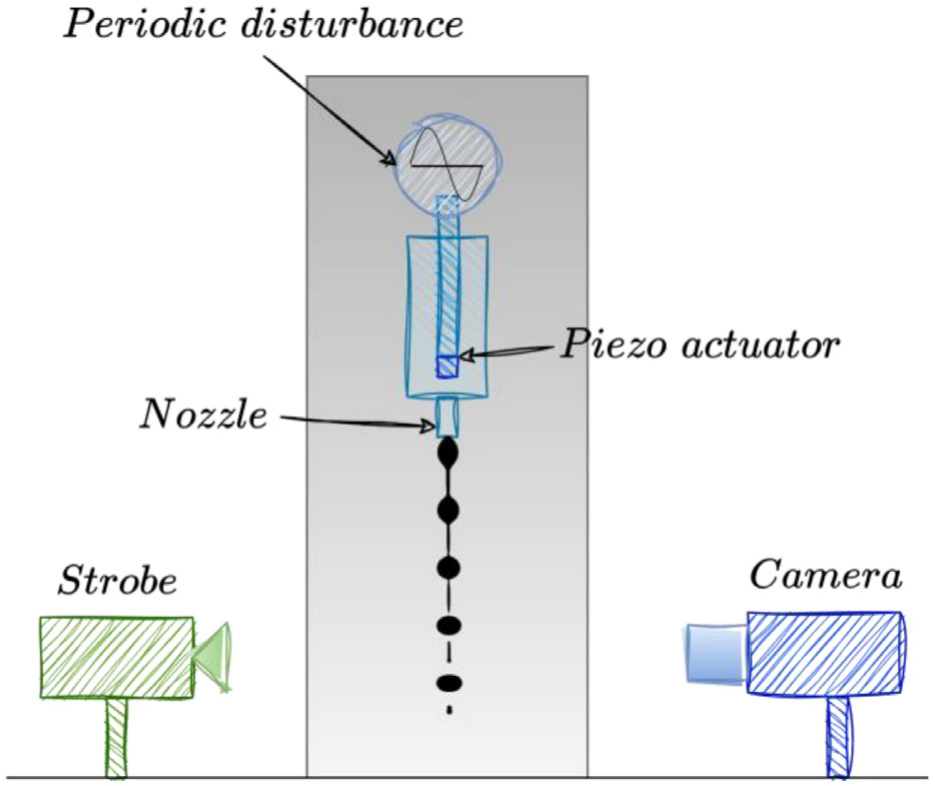
Sketch of the experimental set-up for generating and visualizing drops.

The present project uses a classic CIJ installation which consists in an air-assisted atomization nozzle as depicted Fig. 3.

A HPLC pump is used to deliver the fluid to the print-head via a buffer canister, maintaining a stable flow rate. The fluid is then ejected through the specialized nozzle, and the piezo-actuator, operating at its resonance frequency, introduces controlled disturbances into the fluid stream. Concurrently, the stroboscopic LED panel emits flashes of light at specific intervals, carefully synchronized with the piezo-actuator's frequency.

The Basler acA2040 camera, connected to a computer and controlled through its Python library interface, captures images of the fluid jet breakup illuminated by the synchronized flashes.

The Arduino control system ensures precise coordination between the actuator's frequency and the timing of the flashes. This synchronization allows for the clear and accurate visualization of the Rayleigh-Plateau instability in the jetted fluid with a resolution of 1*px ≈* 1*µm*.

It comprises several key components:

1. Fluid Pump (HPLC 80P Pump from Knauer): The fluid jetting process initiates with a high-performance liquid chromatography (HPLC) pump from Knauer, specifically the HPLC 80P Pump. This pump, equipped with pistons, facilitates precise control of the fluid flow rate.
2. Buffer Canister: Positioned between the pump and the print-head, a buffer canister contains air. This buffer serves to stabilize the fluid flow, ensuring a consistent and steady jet as it exits the nozzle.
3. Nozzle (See Fig. 4): The fluid is jetted through a specialized nozzle, the geometry of which is illustrated in Fig. 4. The nozzle's shape plays a pivotal role in governing the characteristics of the fluid jet and influences the Rayleigh-Plateau instability.

**Fig. 4:**
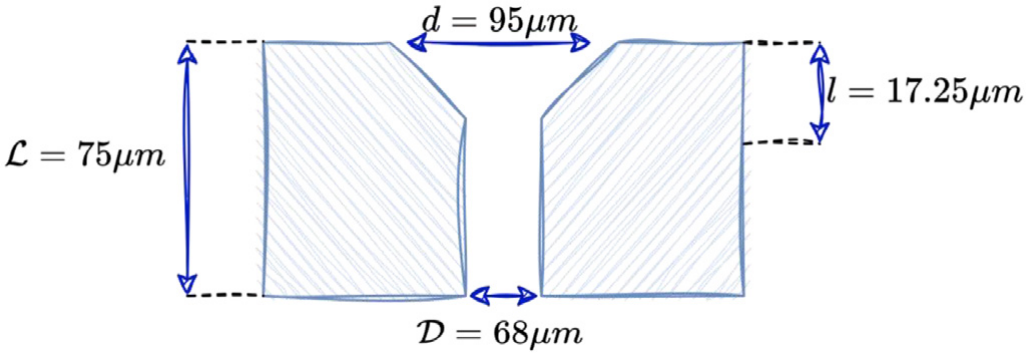
Sketch of a cross-section of the CIJ nozzle, not to scale.
4. Piezo-Actuator: The Rayleigh-Plateau instability in the fluid jet is triggered by a piezo-actuator. Operating at a resonance frequency ranging from 67.2 kHz to 69 kHz, the actuator generates controlled disturbances within the fluid stream, leading to the formation of drops.
5. Stroboscopic Illumination (LED): To capture the breakup dynamics of the fluid jet, stroboscopic illumination is employed. A single flash, synchronized with the piezo-actuator, emits precisely timed flashes of light with a duration of 1 NOP. This synchronized illumination effectively freezes the motion of the fluid jet, facilitating clear and high-speed imaging.
6. Camera (Basler acA2040 Camera): The imaging component of the setup is a Basler acA2040 camera, responsible for capturing images of the fluid jet breakup (see Fig. 2). Controlled by a computer through its Python library interface, the camera records the dynamic behavior of the jet, providing visual data crucial for analysis.
7. Control System (Arduino): Both the piezo-actuator and the stroboscopic LED panel are under the control of an Arduino system. The Arduino board ensures precise synchronization between the actuator's frequency and the timing of the light flashes. This synchronization is essential for accurately capturing the breakup process.

### 4.2 Fluids properties and operating conditions

In this scientific study, our research focused on a set of Newtonian fluids. The fluid selection encompassed deionized water (with a resistivity of 18*.*2*Mohm.cm)* and ethanol as well as dilutions of industrial-grade Emkarox 130 and 681 from Croda, and Polyethylene Glycol (PEG, branded as WSR301 from Polyox) in deionized water. Each fluid underwent a thorough characterization process which involved measuring density and both kinematic and dynamic viscosities using the Lovis 2000 ME Rolling-Ball Viscometer (Anton Paar) with the DMA4500 module, and measuring surface tension using the ring method with an ARES-G2 (Waters) [2].

The fluids properties as well as the operating conditions were listed in Table 1, measurements performed at 23 °C:

**Table 1:** Properties and operating conditions of the jetted fluids in the present dataset.

Fluids								
	Fluid properties				Operating conditions			
Dataset name	Density (*g/cm*^3^)	Kinematic Viscosity (*mm*^2^*/s*)	Dynamic Viscosity (*mPa.s*)	Surface Tension (*mN/m*)	Flow Rate (*ml/min*)	Pressure (bar)	Period (NOP)	Period (*µs*)
EMKAROX130_10%	1.0096	1.714	1.727	42.70	4	4	236	14.75
EMKAROX681_10%	1.0094	2.8593	2.886	41.35	4	4	234	14.625
EMKAROX681_13%	1.0113	3.838	3.882	40.45	4	4	232	14.5
EMKAROX681_17%	1.01659	5.464	5.56	39.89	3.5	4	232-234	14.5-14.625
EMKAROX681_18%	1.01895	6.073	6.188	37.90	3.5	4	232	14.5
Ethanol	0.80996	2.31	1.87	22.26	4	4	236	14.75
Peg_1%	0.99912	1.51	1.51	51.73	4	4	236	14.75
Peg_10-1%	0.99775	0.98	0.98	51.95	4	4	236	14.75
Peg_10-2%	0.9975	0.94	0.93	53.10	4	4	236	14.75
Water	0.99762	0.935	0.933	72.25	4	4	238	14.875

## Limitations

None.

## Ethics Statement

The authors have read and follow the ethical requirements for publication in Data in Brief and confirming that the current work does not involve human subjects, animal experiments, or any data collected from social media platforms.

## CRediT authorship contribution statement

**Guillaume Maîtrejean:** Conceptualization, Methodology, Software, Funding acquisition, Writing – original draft. **Mathis Cousin:** Investigation. **François Truong:** Investigation, Data curation, Software. **Vincent Verdoot:** Investigation, Writing – review & editing. **Frédéric Hugenell:** Investigation, Resources. **Denis C.D. Roux:** Formal analysis, Writing – review & editing.

## Data Availability

Comprehensive Experimental Dataset on Large-Amplitude Rayleigh-Plateau Instability in Continuous InkJet Printing regime (Original data) (Mendeley Data)
